# Investigation of Plasma Metabolic and Lipidomic Characteristics of a Chinese Cohort and a Pilot Study of Renal Cell Carcinoma Biomarker

**DOI:** 10.3389/fonc.2020.01507

**Published:** 2020-08-18

**Authors:** Xiaoyan Liu, Mingxin Zhang, Xiang Liu, Haidan Sun, Zhengguang Guo, Xiaoyue Tang, Zhan Wang, Jing Li, Lu He, Wenli Zhang, Yajie Wang, Hanzhong Li, Lihua Fan, Shirley X. Tsang, Yushi Zhang, Wei Sun

**Affiliations:** ^1^Institute of Basic Medical Sciences, Chinese Academy of Medical Sciences, School of Basic Medicine, Peking Union Medical College, Beijing, China; ^2^Department of Urology, Peking Union Medical College Hospital, Chinese Academy of Medical Science, Beijing, China; ^3^Department of Urology, The Affiliated Hospital of Qingdao University, Qingdao, China; ^4^Beijing Tiantan Hospital, Capital Medical University, Beijing, China; ^5^Core Laboratory for Clinical Medical Research, Beijing Tiantan Hospital, Capital Medical University, Beijing, China; ^6^Principal Investigator BioMatrix Rockville, Rockville, MD, United States

**Keywords:** plasma, metabolomics, lipidomics, renal cell carcinoma, biomarker

## Abstract

Plasma metabolomics and lipidomics have been commonly used for biomarker discovery. Studies in white and Japanese populations suggested that gender and age can affect circulating plasma metabolite profiles; however, the metabolomics characteristics in Chinese population has not been surveyed. In our study, we applied liquid chromatography-mass spectrometry-based approach to analyze Chinese plasma metabolome and lipidome in a cohort of 534 healthy adults (aging from 15 to 79). Fatty-acid metabolism was found to be gender- and age-dependent in Chinese, similar with metabolomics characteristics in Japanese and white populations. Differently, lipids, such as TGs and DGs, were found to be gender-independent in Chinese population. Moreover, nicotinate and nicotinamide metabolism was found to be specifically age-related in Chinese. The application of plasma metabolome and lipidome for renal cell carcinoma diagnosis (143 RCC patients and 34 benign kidney tumor patients) showed good accuracy, with an area under the curve (AUC) of 0.971 for distinction from healthy control, and 0.839 for distinction from the benign. Bile acid metabolism was found to be related to RCC probably combination with intestinal microflora. Definition of the variation and characteristics of Chinese normal plasma metabolome and lipidome might provide a basis for disease biomarker analysis.

## Introduction

Plasma has been widely used for biomarker exploration in various diseases. It is a “data-rich” source that contains several thousands of metabolites and would likely reflect the contributions from various organs. Compared to urine metabolites, plasma metabolites are more stable due to regulation via human homeostasis (1). Lipidomics is a component of plasma metabolomics, which comprehensively analyse the lipid metabolites in the plasma. The definition of the characteristics of the normal plasma metabolome and lipidome would provide a basis for disease analysis, as well as for the understanding of a healthy plasma metabolism state.

Several studies have evaluated plasma metabolomics/lipidomics in healthy subjects based on different ethnic-groups. In 2014, based on white population, Masaki Ishikawa et al. performed lipidomic analyses for fasting plasma and serum samples in 60 healthy adults. The levels of many sphingomyelin species were significantly higher in females than those in males, and the levels of triacylglycerols were significantly higher in elderly females than in young females (2). For U.K population, in 2015 the plasma metabolic phenotyping of 1200 subjects showed that the plasma metabolites were associated with gender and age. Androgen and its derivatives were higher in males, and progesterone and its derivatives were higher in females and were age-associated (3). In 2016, based on 60 healthy Japanese individuals, 516 endogenous metabolites were detected in the sera. Gender-associated differences were found in redox homeostasis and in steroid and purine-nucleotide metabolism pathways. Age-enriched levels of monoacylglycerols were highlighted in Japanese males (4). From the above observations of plasma metabolic characteristics in populations of the white, the UK and Japan, both similarity and differences were observed among different populations. Several studies have suggested that diets and location could influence plasma metabolomics (5, 6), therefore, plasma metabolomics and lipidomics of Chinese population might have different characteristics from population from other countries. An investigation of Chinese plasma metabolites and lipids with respect to age and gender would be an important reference and provide a basis for a biomarker study in the Chinese population. To our knowledge, no such study was available up to now.

Renal cell carcinoma (RCC) is the second most lethal urinary cancer (7). Clinically, the accuracy diagnosis of certain small RCC tumors and for distinction of RCC from benign renal lesions is difficult (8). The development of more accurate and more economical early screening methods might have an important impact on RCC diagnosis. Several studies have applied plasma metabolomics for RCC biomarker discovery. Phenylalanine metabolism, tryptophan metabolism, and arachidonic acid metabolism were found to be related to RCC (9). Deregulated lipid metabolism in RCC has been implicated in disease progression (10, 11). However, few plasma metabolomics and lipidomics study has been used for the distinction of RCC from benign tumors, which would be an important contribution to RCC differential diagnosis.

In present work, we enrolled plasma samples from 534 Chinese volunteers aging from 15 to 79, with a sample size balanced for gender and age. Metabolic and lipidomic characteristics with respect to age and gender in Chinese population were comprehensively investigated. And comparison of Chinese metabolism characteristics with other country population was provided. Further, using plasma metabolomics and lipidomics, RCC potential biomarkers were explored based on age- and gender-matched healthy subjects and RCC patients (143 RCC patients and 34 benign kidney tumor patients). Our study provides an overview of plasma metabolomic characteristics of the Chinese population and a new insight into RCC diagnosis.

## Materials and Methods

### Plasma Collection and Preparation

This study was approved by the Institutional Review Board of the Institute of Basic Medical Sciences, Chinese Academy of Medical Sciences (Approval number: 047-2019). All human subjects, including 534 healthy human adults, 143 RCC patients and 34 benign kidney tumor patients, provided informed consent before participating in this study. The enrolled RCC subjects must meet the following criteria: (1) definite nephroid patients (renal cancer, benign nephrons such as renal cyst, renal angiomyolipoma, etc.) underwent surgery. The control group was the subjects that physical examination index is normal. (2) the functions of the heart, liver, kidney, bone marrow, and other important organs are normal or basically normal. (3) For nephroid patients, ECOG (Eastern Cooperative Oncology Group) score was ranged from 0 to 1. (4) the patient had never received any other anti-renal cancer treatment before. The benign renal tumors in our study include renal angiomyolipoma and renal cysts. All renal cysts were clinically diagnosed according to Bosniak standards. Pathological diagnosis of benign renal cysts was included in the benign renal tumor group. Cystic renal cancers were treated with preservation of the nephron.

Fresh blood of RCC subjects and healthy controls was collected prior to surgery or during physical examination in the morning at 07:00 a.m.−09:00 a.m. after overnight fasting and was subsequently drawn into 10 ml Vacutainer Plasma Separator Tubes with a clot activator for plasma. Next, all samples were centrifuged according to the manufacturer's instructions. Plasma samples were prepared within 2 h after blood collection and stored in a −80°C refrigerator until metabolomics and lipidomics analysis. Metabolomics and lipidomics sample preparation was performed based on the method described in previous references (12, 13) (Supplemental Materials). For plasma sample limitation, only 466 samples were submitted to lipidomics analysis.

### LC-MS Analysis

Metabolomics and lipidomics analyses were conducted using a Waters ACQUITY H-class LC system coupled with an LTQ-Orbitrap mass spectrometer velos (Thermo Fisher Scientific, MA. USA). Detailed information regarding the gradient and MS is provided in Supplemental Materials.

### Data Processing

Raw data files were processed by the Progenesis QI (Version 2.0, Nonlinear Dynamics) software. The detailed processing parameters were provided in the Supplementary Method. Mass list data file exported from QI was further processed by MetaboAnalyst 3.0 (http://www.metaboanalyst.ca), including missing value estimation, log2 transformation and Pareto scaling. Variables missed in 50% or greater of all samples were removed from further statistical analysis and the other missing values were filled using the KNN method. ComBat method was used for batch effect correction. Pattern recognition analysis (principal component analysis, PCA; orthogonal partial least squares discriminant analysis, OPLS-DA) was carried out using SIMCA 14.0 (Umetrics, Sweden) software. Hundred times permutation validation was performed to evaluate the fitting of OPLS-DA model. VIP (Variable importance for the projection) value obtained from OPLS-DA was used for differential metabolites selection. Non-parametric tests (Wilcoxon rank-sum test) were used to evaluate the significance of variables. False discovery rate (FDR) correction (Benjamini method) was used to estimate the chance of false positives and correct for multiple hypothesis testing. Differential metabolites were selected according to the criteria: (1) VIP value above 1; (2) Adjusted p value below 0.05. ROC curve was constructed based on differential metabolites using logistic regression algorithm using MetaboAnalyst 3.0 platform.

### Metabolite Annotation and Pathway Analysis

Metabolites and lipids with adjusted *p* < 0.05 and VIP (for OPLS-DA) >1 were considered as significantly differential features, which would be submitted for further identification. The identification of the significant metabolites based on the Progenesis QI was performed based on the published identification strategy (14, 15) (Supplementary Materials).

Pathway enrichment analysis was performed using MetaAnalyst 3.0 (16) and Mummichog algorithm prediction (17). Mummichog is a program written in Python that leverages the organization of metabolic networks to predict functional pathways directly from feature tables and to generate a list of tentative metabolite annotations through functional activity analysis. The detailed parameters were provided in the Supplementary Materials.

## Results

**Figure 1:**
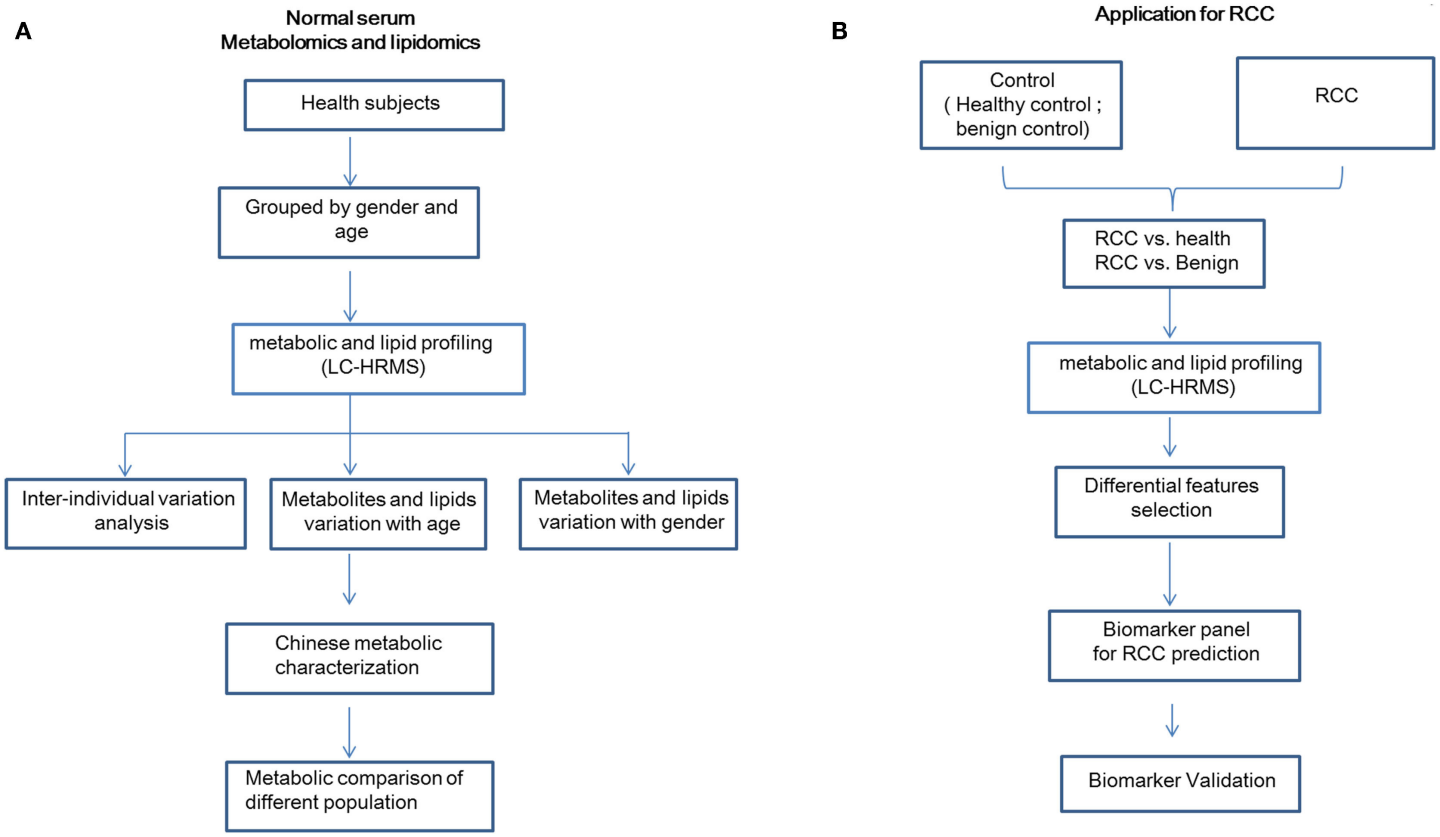
Detailed workflow of this study. **(A)** Workflow for normal plasma metabolomics and lipidomics characterization in a Chinese cohort. **(B)** Workflow for RCC biomarker discovery. This figure was created using Microsoft office PPT 2007.

The detailed workflow of this study is shown in Figure 1. Overall 534 healthy Chinese subjects were enrolled (Table 1). The stability and reproducibility of this study was assessed using quality-control samples (Supplemental Materials).

**Table 1:** Demographics of the enrolled subjects.

	**Male**	**Female**	**Total**
**Healthy subjects**			
Aged 15–30	55	85	140
Aged 31–50	92	146	238
Aged >50	61	95	156
Total	208	326	534
**RCC Patients**			
RCC all (aged 53.3 ± 13.3)	100	43	143
Benign tumor (aged 45.3 ± 11.2)	9	25	34
Healthy control (aged 48.2 ± 13.1)	112	92	204

### Inter-individual Variations of Chinese Plasma Metabolome and Lipidome

**Figure 2:**
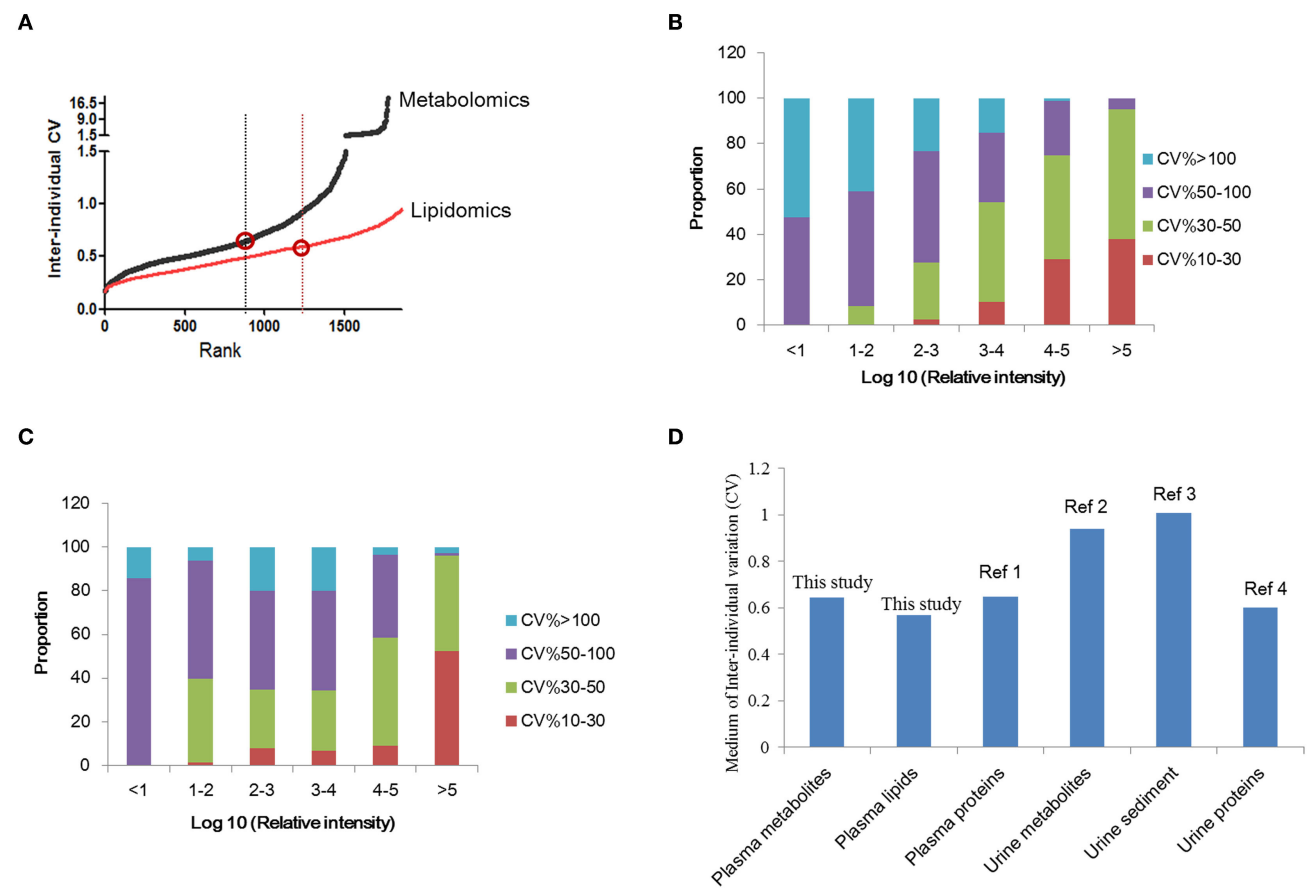
Inter-individual variation analysis of plasma metabolomics and lipidomics. **(A)** Inter-individual variations of plasma metabolomics and lipidomics in normal subjects. The variation of lipidomics is lower than metabolomics. **(B)** The comparison of urine and plasma metabolites/proteins CVs in previous studies and this study. **(C)** Correlation analysis of metabolite CV and abundance in normal subjects. **(D)** Correlation analysis of lipid CV and abundance in normal subjects. This figure was created using Microsoft office Excel 2007. Ref 1: (18); Ref 2: (19); Ref 3: (20); Ref 4: (21).

Determination of the normal inter-individual variations range of metabolites in healthy population would provide baseline reference for future biomarker discovery. A total of 1773 features were quantified in normal plasma metabolome and used for individual variation comparisons. The median of the inter-individual coefficient of variation (CV) for all plasma metabolomes was 0.645 (Figure 2A) (0.645 for females and 0.626 for males). For age variation, the CV was about 0.6 (Figure S1). For lipidomics, overall, 2239 features were quantified. The median CV for all plasma lipidomes was 0.568 (Figure 2A). Similar to the results of metabolomics, the lipidome CV of males is lower than that of females. In contrast to metabolomics, the lipidomic CV increased with aging; however, it showed no significance (Figure S1).

Further, the relationship between metabolite abundance and inter individual variation (CV) was analyzed. The results showed that features with high intensity always showed lower CVs. CVs of more than 90% high-abundance features ranged from 10 to 50%. Features with low abundance showed higher CVs, always over 50%. These results indicated the higher stability of metabolites with high abundance (Figure 2B). The same trend was observed for plasma lipids (Figure 2C).

Herein, we compared the inter-individual variations of plasma metabolite/lipid/protein with previously reported urine results (Figure 2D) (19–21). It is suggested that the variation of plasma metabolomics and lipidomics were lower than that for urine metabolites. And plasma proteins variation, reported the medium as 0.67 (18), was similar to plasma metabolite variation. Comparison of plasma and urine metabolites/protein variations indicated that plasma metabolites/proteins are more stable than urine metabolites/proteins.

### Chinese Plasma Metabolomics and Lipidomics Are Gender-Dependent

**Figure 3:**
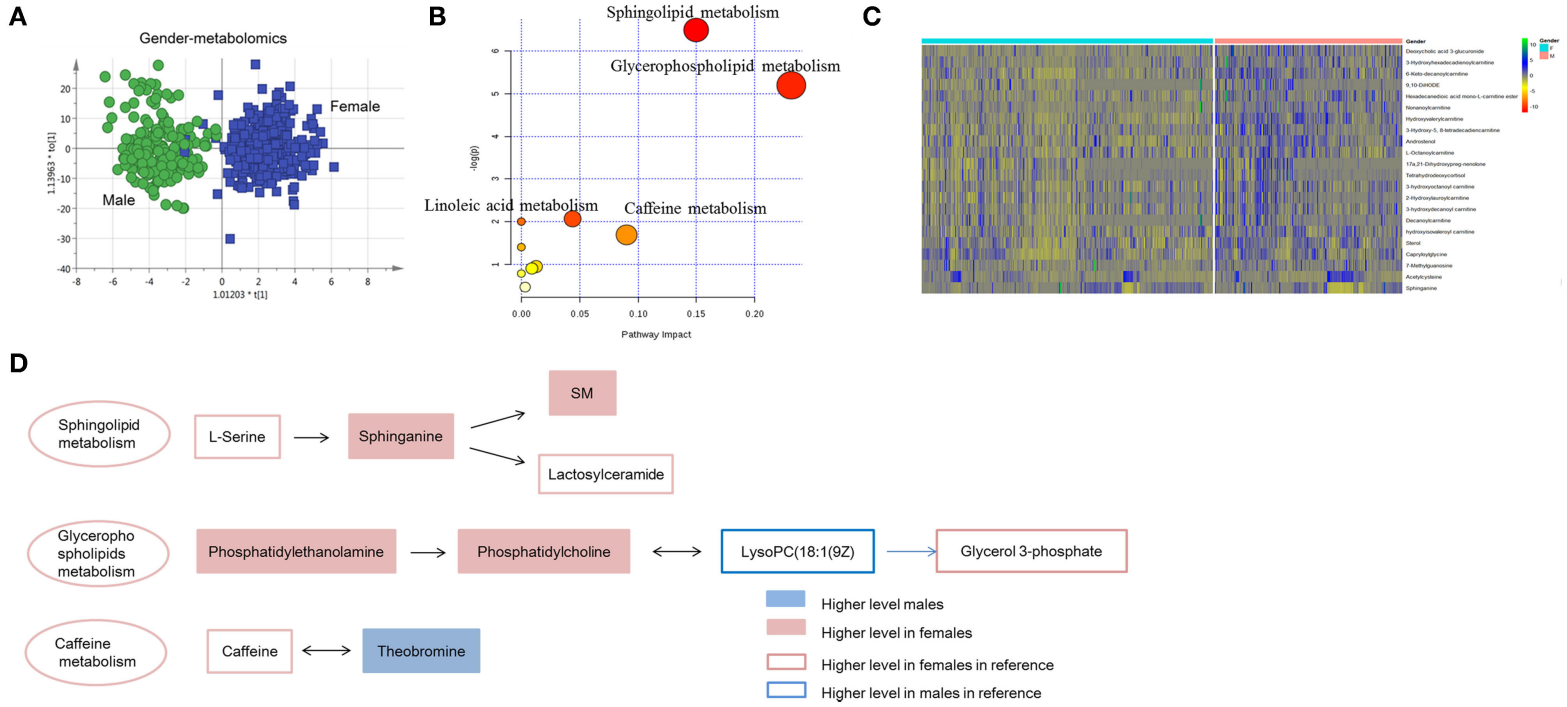
Gender-dependent metabolomics/lipidomics characteristics. **(A)** Score plot of OPLS-DA model between females and males. Apparent separation of metabolomics was showed between males and females. **(B)** Pathway enrichment of gender-dependent metabolites and lipids. **(C)** Heat map showing the relative abundance of gender-dependent metabolites/lipids these performing important biological functions. **(D)** Highlighted pathways contain specific metabolites showing differences between the male and female. **(A)** Was created using simca 14.0; **(B)** Was created using MetaboAnalyst 3.0; **(C)** Was created using R package; **(D)** Was created using Microsoft office PPT 2007.

To explore the metabolites contributing to gender discrimination, unsupervised PCA showed differential tendency of the plasma metabolic profile between males and females (Figure S2A). OPLS-DA model was further used for differential feature selection (Figure 3A and Figure S2B). Overall 27 differential metabolites contributing to gender discrimination were identified (Table S1). All these metabolites except sphinganine showed a higher level in males, including acylcarnitines, steroids and acyl-amino acids (Figure S2C). Using the same strategy, a total of 160 gender-differential lipids was identified (Figure S3 and Table S2). In females, phospholipidswere found to be higher. While steroids, fatty acids, acylcarnitines and DG showed higher level in males (Figure S3D).

Gender-dependent metabolites and lipids were further submitted to pathway enrichment. Sphingolipid metabolism, glycerophospholipid metabolism, caffeine metabolism and linoleic acid metabolism showed differences between males and females (Figure 3B). Several important metabolites/lipids involved in these pathways, such as sphinganine, PE, PC and theobromine have been reported in previous studies (2–4) and probably showed important roles in gender-differential biological functions (Figure 3C and Table S3). Sphingolipid metabolism showed more active in females than males. The upstream metabolites, serine has been reported showing higher level in females (3). Consequently, the down-stream metabolites, sphinganine, SM and lactosylceramide showed higher level in females. Metabolites in glycerophospholipids metabolism showed inconsistent trend in male and female. Phosphatidylethanolamine, phosphatidylcholine and the downstream metabolites, glycerol 3-phosphate showed higher level in females. While the intermediate metabolites, LysoPC(18:1(9Z) has been reported to be higher in males, which was probably regulated by other pathways (3). Caffeine metabolites, teobromine was found to be higher in males, probably the consequence of increased caffeine metabolism in males (Figure 3D).

### Chinese Plasma Metabolomics and Lipidomics Are Age-Dependent

**Figure 4:**
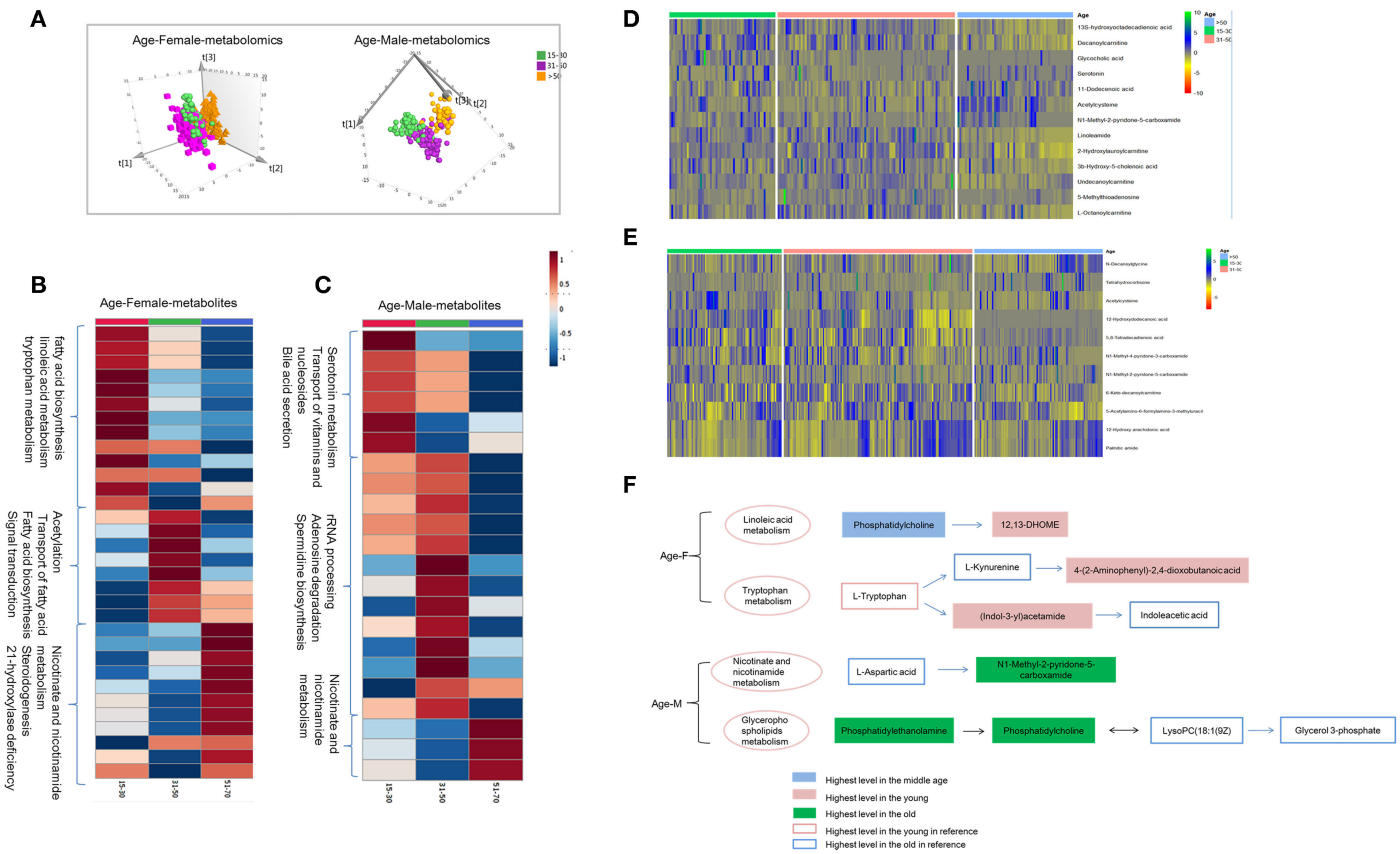
Age-dependent metabolomics/lipidomics characteristics. **(A)** Score plot of PLS-DA model for three age groups based on plasma metabolomics in females and males. Metabolic profiling varied with age in both males and females. Differential metabolites and pathways with respect to aging in male **(B)** and female **(C)**. Heat maps showing the relative abundance of age-dependent metabolites/lipids in male **(D)** and female **(E)** these performing important biological functions. **(F)** Highlighted pathways contain specific metabolites showing differences with age in male and female. **(A)** Was created using simca 14.0; **(B,C)** Were created using MetaboAnalyst 3.0; **(D,E)** Were created using R package; **(F)** Was created using Microsoft office PPT 2007.

Age is another non-negligible factor for metabolomics research. Herein, we respectively examined metabolomics and lipidomics patterns with age for males and females, respectively. Unsupervised PCA score plot showed slight separation trend of three age groups for male and female (Figure S4). Supervised PLS-DA score plot presents the apparent discrimination of three age groups for both females and males (Figure 4A and Figures S5A,B). In young females, metabolites of 3-indolebutyric acid, 16-hydroxy-10-oxohexadecanoic acid, and indole-3-acetamide showed the highest level in the young group (Figure 4B and Table S4), which suggested that fatty-acid biosynthesis, linoleic-acid metabolism, and tryptophan metabolism were more active. In contrast, in young males, the processes associated with serotonin metabolites, transport of vitamins and nucleosides, and bile-acid secretion were activated (Figure 4C and Table S5). Additionally, in the middle and the old age, male and female showed different metabolism and lipidomic characteristics (Tables S6, S7 and Figure 4, Figures S5C,D).

Combination analysis of age-dependent metabolites and lipids showed enriched pathways these varied with age in female and male. Several metabolites and lipids have been reported in previous researches (2–4) and play important roles in age-differential biological functions (Figures 4D,E and Table S3). In female, linoleic acid metabolism and tryptophan metabolism were found to be changing with age. Linoleic acid metabolites, 12,13-DHOME showed higher level in the young female. While the upstream metabolites, phosphatidylcholine showed lower level. Increased phosphatidylcholine metabolism probably lead to 12,13-DHOME accumulation in young females. As a whole, tryptophan metabolites showed higher activity in the young females. Higher level of (Indol-3-yl) acetamide probably results from the higher tryptophan metabolism and lower (Indol-3-yl) acetamide metabolism in young females (Figure 4F). In males, nicotinate and nicotinamide metabolism and glycerophospholipids metabolism showed age dependent, showing higher level in the old male population. Age dependent metabolites obtained in present study and previous references showed a consistent change trend (Figure 4F).

### Plasma Metabolomics and Lipidomics Distinct RCC From Healthy and Benign Controls

**Figure 5:**
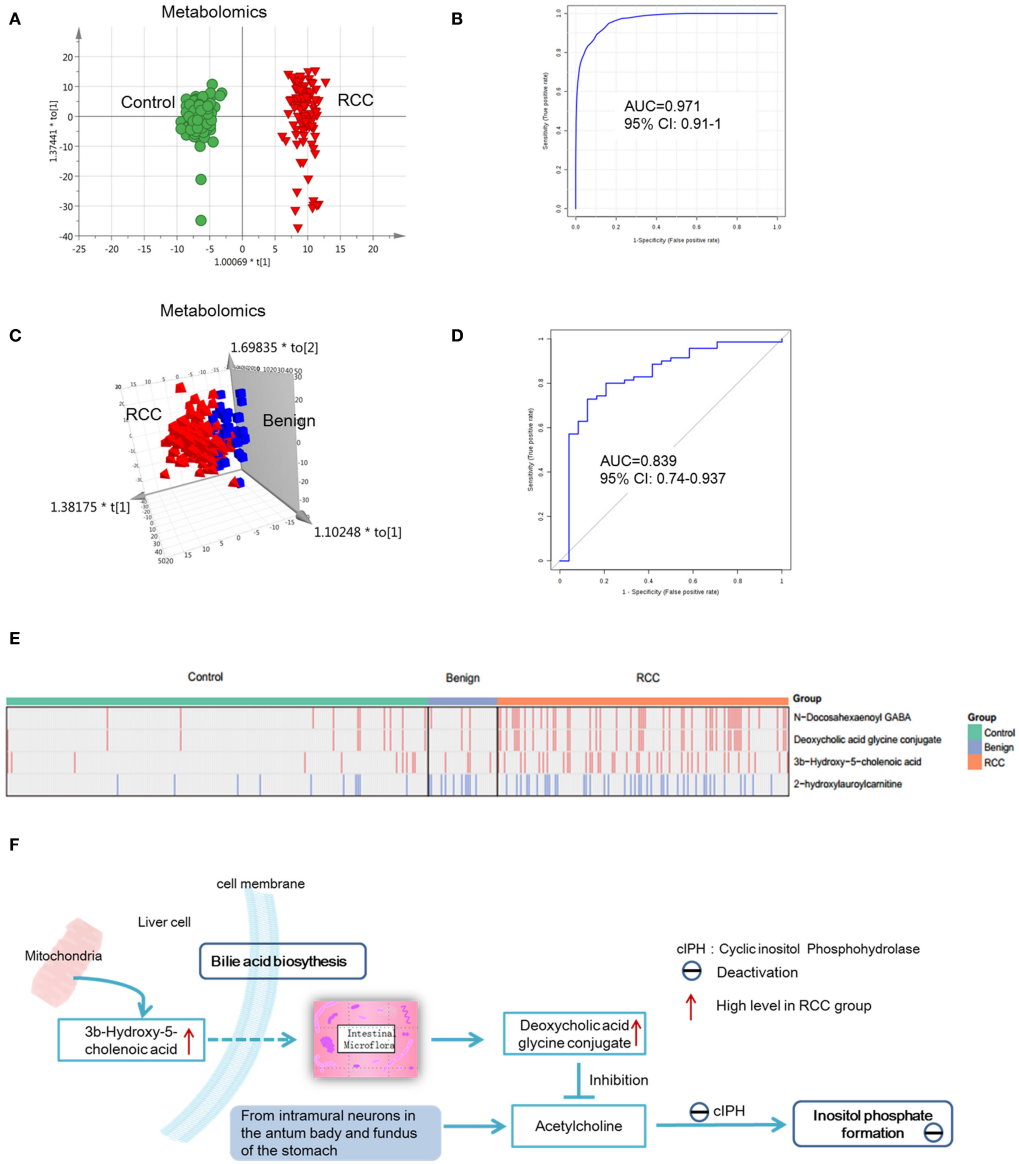
Analysis of metabolite and lipid differences between RCC, control and benign tumors. **(A)** OPLS-DA-score plot of serum metabolomics between RCC and healthy control. The apparent separation indicated significant differences of metabolites between RCC and control groups. **(B)** ROC plot for RCC discrimination from control in validation group based on model established via differential metabolites and lipids through a panel consisting of diaminopimelic acid, 12,13-DHOME, 5-L-glutamyl-L-alanine, PC(38:4), 4,8-dimethylnonanoyl carnitine and cholesteryl 11-hydroperoxy-eicosatetraenoate. **(C)** OPLS-DA-score plot of serum metabolomics between RCC and benign tumors. Significant differences of metabolites and lipids between RCC and benign groups were existed. **(D)** ROC plot for RCC discrimination from benign tumors with 10-fold cross-validation based on the model established via differential metabolites and lipids, L-glutamine, PS(36:0), PG(40:9), N-docosahexaenoyl GABA and deoxycholic acid glycine conjugate. **(E)** Relative intensity of the common four metabolites/lipids in RCC and non-RCC groups. Take the four metabolites/lipids with a concentration above the 90% quantile of normal range in healthy population as positively upregulated/downregulated, they all showed higher prevalence in renal cancer patients than healthy controls and benign patients. Red: increased; blue, decreased. **(F)** Interaction of differential metabolites of RCC and the possible regulation mechanism in RCC. Dotted arrow, indirect action; solid arrow, direct action. **(A,C)** Were created using simca 14.0; **(B,D)** Were created using MetaboAnalyst 3.0; **(E)** Was created using R package; **(F)** Was created using Microsoft office PPT 2007.

To discover potential biomarkers for distinction of RCC and healthy controls, all subjects were divided into a discovery group (98 RCC vs. 135 control) and a validation group (45 RCC and 69 control). PCA was first performed to explore metabolic and lipidomic profiling variations between RCC and control in the discovery dataset. The score plot showed discrimination trend (Figures S6A,B). Apparent separation was further visualized via a score plot of OPLS-DA (Figure 5A and Figure S6C). Overall, 19 differential metabolites and 11 lipids were identified that contributed to discrimination among the groups (Table 2).

**Table 2:** Differential metabolites and lipids between RCC and health control groups.

**Compound ID**	**Description**	**Score**	**VIP**	***P-value***	**Fold change(RCC/Control)**
**Metabolites**					
HMDB31057	(R)-2-Hydroxyhexadecanoic acid	40.8	1.85	9.10E-02	0.41
HMDB04705	12,13-DHOME	48.4	2.05	2.23E-02	0.31
HMDB29998	12-Hydroxy-8,10-octadecadienoic acid	38.5	1.43	5.36E-02	0.51
HMDB60043	13-HDoHE	40.1	1.34	7.92E-17	1.97
HMDB41287	16-Hydroxy-10-oxohexadecanoic acid	49.1	3.16	1.47E-10	0.11
HMDB31103	2-Hydroxylinolenic acid	38.5	3.25	3.57E-05	0.13
HMDB00308	3b-Hydroxy-5-cholenoic acid	43.7	1.52	3.96E-03	2.58
HMDB61636	3-hydroxydecanoyl carnitine	42.3	1.33	1.28E-17	0.56
HMDB61634	3-hydroxyoctanoyl carnitine	46.8	1.23	9.56E-31	0.60
HMDB06248	5-L-Glutamyl-L-alanine	53.8	2.08	3.94E-18	0.37
HMDB04710	9,10,13-TriHOME	45.6	4.05	1.16E-11	0.05
HMDB00631	Deoxycholic acid glycine conjugate	54.8	2.19	2.33E-09	3.37
HMDB00157	Hypoxanthine	40.8	1.60	2.44E-44	0.43
HMDB34125	Licoisoflavone A	35.9	4.93	6.21E-27	33.05
HMDB62332	N-Docosahexaenoyl GABA	41.4	2.24	9.83E-05	3.78
HMDB39500	N-Malonyltryptophan	39.2	2.21	2.30E-23	3.57
HMDB62631	O-decanoyl-L-carnitine	52.6	1.14	1.76E-18	0.65
HMDB32596	Sodium glycocholate	42.8	1.36	4.63E-09	1.72
HMDB13321	Undecanoylcarnitine	52	1.56	1.94E-13	0.31
**Lipids**					
LMFA07070032	2-Hydroxylauroylcarnitine	43.8	1.12	7.78E-04	0.70
LMFA07070046	4,8-dimethylnonanoyl carnitine	44.2	2.54	6.78E-12	0.33
49703674	Cholesteryl 11-hydroperoxy-eicosatetraenoate	38.6	3.41	1.32E-15	0.27
123060174	delta2-THA	48.8	1.48	1.20E-08	0.37
135638187	DG(32:0)	48.4	1.26	2.11E-04	1.51
7849711	Isobehenic acid	48.9	2.56	2.42E-10	0.34
7850526	N-arachidonoyl glycine	43.3	1.17	5.45E-06	0.53
123067362	PC(38:4)	42.1	2.35	3.28E-11	2.22
74380445	PS(18:0)	45.8	1.50	1.51E-09	1.63
123063109	PS(38:4)	41.1	1.03	4.29E-03	1.45
4266327	Vitamin K2	39	1.57	3.43E-04	0.62

Predictive models based on differential metabolites, lipids, or metabolites-lipids combination were constructed. It showed a better discrimination accuracy using metabolites-lipids combination than only metabolites or lipids. A metabolites-lipids panel consisting of diaminopimelic acid, 12,13-DHOME, 5-L-glutamyl-L-alanine, PC (38:4), 4,8-dimethylnonanoyl carnitine and cholesteryl 11-hydroperoxy-eicosatetraenoate achieved an ROC area of 0.996 for the testing dataset and 0.971 for the external validation dataset (Figure 5B), which is higher that AUC values obtained only using metabolites (0.991 for testing set; 0.987 for validation set) or lipids (0.958 for testing set; 0.841 for validation set) (Table 3).

**Table 3:** Performance of metabolites/lipids panel for group discrimination.

**ROC analysis**	**Plasma metabolomics**		**Plasma lipidomics**		**Combined plasma metabolomics and lipidomics**	
Groups	Discovery group	Validation group (cross-validation)	Discovery group	Validation group	Discovery group	Validation group (cross-validation) Validation group
RCC vs. Control	0.991	0.987	0.958	0.841	0.996	0.971
RCC vs. Benign tumor	0.811	0.776	0.839	0.785	0.898	0.839

Using the same analysis strategy, significant separation was observed between RCC and benign metabolite and lipid profiling (Figure 5C). Overall 8 differential plasma metabolites and 15 lipids were identified (Table 4). Metabolites of L-glutamine and deoxycholic acid glycine conjugate and lipids of vitamin D3 metabolites, fatty acyl carnitines, PS, PE and DG were upregulated in the RCC group. Differential diagnosis accuracy for RCC was evaluated using ROC analysis. Similarly, metabolite-lipid combination could achieve better discrimination accuracy for RCC and benign (Table 3). A metabolite-lipid panel consisting of L-glutamine, PS(36:0), PG(40:9), N-docosahexaenoyl GABA and deoxycholic acid glycine conjugate showed the best predictive ability with an ROC area of 0.898 for the testing dataset and 0.839 for 10-fold cross-validation (Figure 5D and Table 3).

**Table 4:** Differential metabolites and lipids between RCC and benign groups.

**Compound ID**	**Description**	**Score**	**VIP**	***P-value***	**Fold change (RCC/benign)**
**Metabolites**					
HMDB00308	3b-Hydroxy-5-cholenoic acid	46.8	4.69	4.43E-02	2.36
HMDB02596	Deoxycholic acid 3-glucuronide	53.4	3.17	8.17E-03	3.33
HMDB00631	Deoxycholic acid glycine conjugate	55.1	4.92	1.35E-02	2.42
HMDB02579	Glycochenodeoxycholic acid 3-glucuronide	51.1	3.51	1.06E-02	7.48
HMDB00641	L-Glutamine	44.8	2.38	1.31E-02	2.08
HMDB62332	N-Docosahexaenoyl GABA	42.8	4.70	1.72E-02	2.29
HMDB32596	Sodium glycocholate	43.7	3.58	1.90E-02	2.71
HMDB60117	Tetracosahexaenoic acid	39.7	3.78	3.80E-02	2.66
**Lipids**					
24702281	17-Methyltestosterone	37.1	1.47	3.95E-02	0.60
LMFA07070032	2-Hydroxylauroylcarnitine	45.1	1.61	1.70E-02	1.61
LMFA07070033	2-Hydroxymyristoylcarnitine	42.8	1.83	4.13E-02	1.70
135638093	DG(29:2)	41.8	1.05	4.15E-02	1.58
14710962	DG(38:4)	41.5	1.31	4.15E-02	1.40
74380326	N-arachidonoyl tyrosine	42.9	1.41	4.90E-02	1.92
160779918	PC(40:4)	45.8	1.13	4.90E-02	1.24
123061482	PC(O-20:0/22:4)	50	1.15	4.15E-02	1.21
123062511	PE(O-16:0/13:0)	37.6	1.10	4.90E-02	1.39
123067375	PE(P-18:0/20:4)	46.8	1.10	4.15E-02	0.81
123064120	PG(36:3)	53	1.05	4.41E-02	0.68
123064655	PG(40:9)	37	1.58	1.63E-02	1.73
123063586	PS(36:0)	50.6	1.24	4.98E-02	1.45
123063234	PS(38:0)	48	1.21	4.90E-02	0.40
123063371	PS(38:1)	41.5	1.15	4.90E-02	1.38

Common differential metabolites or lipids between RCC vs. control and RCC vs. benign would indicate the specific potential biomarkers for RCC distinction from nonRCC. Herein, 3 metabolites and 1 lipid, deoxycholic acid glycine conjugate, N-docosahexaenoyl GABA, 3β-hydroxy-5-cholenoic acid and 2-hydroxylauroylcarnitine, were found to be common changed. The former three metabolites showed upregulated level, and 2-hydroxylauroylcarnitine showed downregulated level in RCC group. Take the four metabolites/lipids with a concentration above the 90% quantile of normal range in healthy population as positively upregulated/downregulated, they all showed higher prevalence in renal cancer patients than healthy controls and benign patients (Figure 5E). The accuracy for RCC distinction from nonRCC using the four metabolites was assessed using ROC plots. The AUC values were above 0.7 for all those four metabolites (Table 5). Combination of bile acids metabolites, 3β-hydroxy-5-cholenoic acid and deoxycholic acid glycine conjugate could achieve a better discrimination accuracy with the AUC of 0.79.

**Table 5:** Prediction accuracy of metabolites for distinction RCC from non-RCC.

**Metabolites**	**AUC**	**Sensitivity**	**Specificity**
N-Docosahexaenoyl GABA	0.84	0.8	0.9
3b-Hydroxy-5-cholenoic acida	0.76	0.8	0.7
Deoxycholic acid glycine conjugateb	0.75	0.8	0.6
2-hydroxylauroylcarnitine	0.70	0.8	0.5
Combination of metabolites “a” and “b”	0.79	0.7	0.8

## Discussion

In the present study, we performed a comprehensive metabolomics and lipidomic analysis of blood from healthy adults. We demonstrated that age and gender have substantial effects on global plasma metabolite profiles in a Chinese cohort, which showed differences with cohorts from other countries. We also identified pathways associated with gender and age in the Chinese population. Several highlighted pathways present gender-associated differences: sphingolipid metabolism, glycerophospholipid metabolism, caffeine metabolism, and linoleic acid metabolism.

While tryptophan metabolism, nicotinate, and nicotinamide metabolism and glycerophospholipids metabolism present age-associated differences. Further application for RCC biomarker discovery was performed, showing potential value of plasma metabolomics for RCC diagnosis and mechanism exploration.

### Inter-individual Variation of Plasma Metabolomics and Lipidomics

Inter-individual variations of the metabolite/lipid/protein levels are critical factors for designing studies on the exploration of biomarker candidates, as large inter-individual variations in healthy states might mask the changes in metabolite/lipid/protein levels in response to diseases (22). Genetics, sex, age, gastrointestinal flora and lifestyle differences are likely to be important factors (23). Herein, we compared Inter-individual variations of plasma omics and urine omics. As a whole, plasma metabolites/proteins showed lower variation than urine metabolites/proteins. Homeostasis regulation in blood may account for the results (19). Urine metabolites are easily affected by environmental factors, such as water and diets intake (24). The inter-individual variations of plasma metabolites, lipids, and proteins are pretty close, around 0.6, indicating the stability of plasma omics for biomarker research.

### Gender-Dependent Plasma Metabolic Characteristics for Chinese Population

Gender-associated differences of urine metabolites have been characterized in Chinese populations (19). Similarly, several common gender-dependent metabolic features were observed in the urine and plasma. Acylcarnitines, including 6-keto-decanoylcarnitine, non-anoylcarnitine, L-octanoylcarnitine, and steroids, including androstenol, tetrahydrodeoxycortisol, and sterol, were higher in males than in females both in the urine and plasma, indicated a more active steroid-hormone biosynthesis and fatty-acid oxidation in males (25–27). In addition, our results indicated that gender influences the levels of sphingolipids and glycerophospholipids in plasma and, therefore, are confounding factors in exploring lipid biomarkers. Many of sphingolipids and glycerophospholipids are female-enriched in Chinese in this study, which is consistent with Caucasian populations (22). It has been reported that plasma sphingolipids and glycerophospholipids levels are affected by different lifestyles (28). And fruit and vegetable intake could influence sphingolipid and glycerophospholipids levels with significant genetic contributions (29). Therefore, food preference and genetic factors might contribute to gender-dependent differences of sphingolipid and glycerophospholipids. The exact mechanisms of the regulation of lipid metabolism have not been elucidated. A previous study suggested that estrogen may be involved in the regulation of lipid metabolism, such as SM and DHA-containing phospholipids (PLs) (30, 31).

### Age-Dependent Plasma Metabolic Characteristics for Chinese Population

Previous studies had reported that age influences the levels of several metabolites in blood and therefore are confounding factors in exploring disease biomarkers (32). Herein, we explored the biological relevance of age-dependent metabolites based on 534 healthy subjects aged 15–70 years, which may be representative of the general aging population. Our results suggested that linoleic acid metabolism and tryptophan metabolism was more active in young females, which is consistent with our previous urine metabolomics study (19). Increased fatty-acid metabolism may indicate enhanced function of mitochondria in the young (33). The nicotinate and nicotinamide metabolites, N1-methyl-2-pyridone-5-carboxamide (2PY) and N1-methyl-4-pyridone-3-carboxamide showed higher level in old males and females. The same change trend was observed for 2PY in a previous study (34). The upstream metabolites, aspartic acid has been reported showing higher level in the elder population (3), which is consistent with our results. Age-dependent nicotinate and nicotinamide metabolism alterations were probably a consequence of both decreased renal excretion and increased production in the liver or other organs with aging. Additionally, glycerophospholipids, including PE and PC showed higher level in the old males, probably associated with food preferences and metabolism ability weakness in the old (29).

### Comparison of Chinese Metabolic Characteristics With Different Countries

Our study provided an overview of the metabolomics characteristics in a Chinese population. The comparison of gender- and age-dependent metabolites in the Chinese population with that in other populations would provide insight into the metabolic characteristics of Chinese.

Fatty-acid metabolism was found to be gender- and age-dependent in Chinese, Japanese and white populations, probably resulting from the common gender- and age-differential energy metabolism characteristics, regardless of the backgrounds [6, 8]. Additionally, some unique metabolic characteristics were found in different populations. Large molecular lipids, such as TGs and DGs, were found to be gender-associated in whites [6, 7], but they were not significant in Chinese or Japanese. It was reported that TGs synthesis and lipid metabolism showed racial differences, with Caucasians having higher TG synthesis as compared with African-Americans (35). Additionally, preferences for high-lipid diets, such as hamburgers, among white people could partly contribute to these changes. In the present Chinese study, nicotinate and nicotinamide metabolism was found to be specifically age-related. Nicotinate metabolism shows racial differences in African Americans compared to Whites, primarily due to differences in CYP2A6 enzyme activity (36). The genetic differences could contribute to metabolism differences of Chinese and other populations. The above data showed that differences in genetic background and diets could both probably contribute to the metabolism differences of Chinese population compared to other populations.

### Plasma Metabolic Characteristics of RCC

Since gender and age are important confounders for plasma metabolomics and lipidomics, RCC biomarker analysis was explored based on age- and gender-matched control and RCC subjects. Combination of metabolites and lipids could achieve better distinction of RCC from the healthy control and the benign, with AUC of 0.971 and 0.839, respectively.

It was reported that antioxidant defense mechanism was occurred in RCC (37, 38). In present study, we found several disordered pathways in RCC, including fatty acid oxidation, glutamine metabolism, and glycerophospholipids metabolism. These metabolite variations were probably resulting from antioxidant defense mechanism.

Fatty acyl carnitines showed disordered level in RCC group, indicating fatty acid oxidation dysfunction (38). Acylcarnitines are essential for the entry of fatty acid into the mitochondria for oxidation. Carnitine palmitoyltransferase 1 A (CPT1A), a key enzyme in fatty acid oxidation, may contribute to changes of acylcarnitines. CPT1A is a direct HIF (hypoxia-inducible factor) target gene. CPT1A is repressed by HIF1 and HIF2 in RCC, reducing fatty acid transport into the mitochondria, and forcing fatty acids to lipid droplets for storage (39). Our findings are consistent with previous reports, that fatty acid oxidation might be an important factor in determining cancer status.

Glutamine showed increased level in RCC group, which was consistent with previous research (38, 40). In ccRCC, glutamine is subjected to reductive carboxylation leading to production of the onco-metabolite 2-HG (41), as well as being a precursor for the major antioxidant system comprised of GSH and GSSG (7). Blocking glutamate production from glutamine by GLS inhibition will down-regulate this important antioxidant pathway, resulting in higher ROS levels, which will be selectively toxic to cancer cells due to their increased local ROS levels (42). Above data showed the importance of glutamine metabolic pathway to ccRCC.

Glycerophospholipids changes in RCC group was probably associated with disordered oxidative phosphorylation (43, 44) and substrates, choline level changes (40). Fatty acid compositions in PC have been reported to be significantly differential between the normal and RCC tissues, which was resulting from expression variations of lipidomic genes that encode proteins involved in fatty acid elongation, including *SCD, ELOVL5* and *FADS1* (45). Additionally, the content of PE was found to be differential between RCC and controls. PE synthesis was downregulated in ccRCC (45). Disorder of PE synthesis may lead to tumor development by accelerating cell proliferation (46). The underlying mechanism by which PE abundance regulates the proliferation of renal cancer cells remains to be further elucidated.

Bile acid metabolites, 3b-Hydroxy-5-cholenoic acid and deoxycholic acid glycine conjugate and neurotransmitter metabolites, N-docosahexaenoyl GABA showed significantly increased in RCC, compared with control and benign groups. These metabolites have potential value for RCC distinction from non-RCC. It was reported that bile acids were important for renal pathophysiology by activating nuclear receptor farnesoid X receptor (FXR) and the membrane-bound G protein-coupled bile acid receptor 1 (GPBAR1, also known as TGR5) (47). And these receptors were found to be associated with RCC pathogenesis. It was reported that TGR5 could inhibit inflammation by inhibiting the NF-κB signaling pathway, eventually attenuating diabetic nephropathy (DN) (48). And in RCC, it could protect against renal inflammation and renal cancer cell proliferation and migration (49). FXR is a pivotal factor in cholesterol/bile acid homeostasis, and FXR could stimulate proliferation of renal adenocarcinoma cells (50). Moreover, TCGA gene data showed disordered expression level of TGR5 and FXR in RCC tissue, further providing evidence on important roles of these receptors during RCC (37). In this study, we found increased level of bile acid metabolites in RCC group, further proved that bile acid metabolism was disordered in RCC. Another mechanism of bile acid conjugate changes in RCC is possibly associated with micro flora and inositol phosphate pathway (Figure 5F). Deoxycholic acid glycine conjugate is produced by the intestinal micro flora. It has been reported that deoxycholic acid glycine conjugate could inhibit acetylcholine-induced inositol phosphate formation (51). Inositol phosphate formation is catalyzed by cyclic inositol phosphohydrolase, whose activity is reduced in human renal tumor (52). Probably, cyclic inositol phosphohydrolase is the target of acetylcholine. And the increased secretion level of deoxycholic acid glycine conjugate in RCC patients inhibited cyclic inositol phosphohydrolase activity.

Previous study found neuro-effect was involved in RCC occurrence (53). In this study we found that N-docosahexaenoyl GABA showed significantly differential level between RCC and control groups. It is a gamma amino acid (GABA) derivative. GABA is an inhibitory neurotransmitter found in the nervous systems. It acts by binding to specific transmembrane receptors in the plasma membrane of both pre- and postsynaptic neurons. It was reported that GABA A receptor subunitθ (GABRQ) could serve as a novel prognostic marker of ccRCC. Low GABRQ expression was associated with a poor prognosis among patients with ccRCC (54). And, TCGA group discovered significant differences of GABRQ expression between RCC tissues and controls (37). Above findings indicated potential roles of GABA in RCC. GABA metabolites, N-docosahexaenoyl GABA has been shown to have anti-inflammatory, anti-nociceptive, vasoprotective, angiogenic and neuroprotective effects (55). In this study, the increased plasma concentration of N-docosahexaenoyl GABA might be due to the metabolism disorder of GABA. And the relationship of GABA metabolites and GABA receptors need further investigation.

## Conclusion

In conclusion, this study conducted a comprehensive characterization of the plasma metabolome and lipidome in a large cohort of the healthy Chinese population. The results demonstrated gender and age are two important influencing factors of the plasma metabolome and lipidome. Sphingolipids and glycerophospholipids were found to be female-enriched. Linoleic acid metabolism, tryptophan metabolism and nicotinate and nicotinamide metabolism were age dependent in Chinese. These results suggested age and gender matches or corrections were necessary during differential analysis. Using age and gender-matched healthy subjects, a pilot biomarker study on RCC was performed. It showed satisfactory prediction accuracy for RCC using plasma metabolite panels. Antioxidant defense mechanisms might contribute to lipids or oxidative metabolites variations in RCC. Additionally, bile acid metabolites and neurotransmitter, GABA metabolites were found to be associated with RCC occurrence.

Except gender and age, diets and circadian rhythm could also influence plasma metabolomic. For future analysis, we would analyse these factor using diet standardization design. Additionally, present RCC group included multiple subtypes, including clear cell renal cell carcinoma (81%), chromophobe renal carcinoma and other rare types (19%), which could cause interference to metabolomics results. Future analysis on a larger sample size for one specific RCC subtype would be performed to eliminate the inferences.

## Data Availability Statement

The datasets presented in this study can be found in online repositories. The names of the repository/repositories and accession number(s) can be found below: PeptideAtlas, (PASS01608) (http://www.peptideatlas.org/PASS/PASS01608).

## Ethics Statement

This study was approved by the Institutional Review Board of the Institute of Basic Medical Sciences, Chinese Academy of Medical Sciences. The patients/participants provided their written informed consent to participate in this study.

## Author Contributions

XiaoL interpret the data and wrote the manuscript. MZ designed the experiments. WS and YZ conceived and designed the study, helped to interpret the data and revised the manuscript. XianL performed the experiment. ZG and HS helped to perform data analysis. XT, JL, and ZW collected the RCC samples. LH, WZ, and YW collected the health samples. HL helped to revise the manuscript. LF and ST helped to revised the language. WS is the guarantor of this work and, as such, had full access to all the data in the study and takes responsibility for the integrity of the data and the accuracy of the data analysis. All authors contributed to the article and approved the submitted version.

## Conflict of Interest

ST was employed by BioMatrix, Rockville, Maryland, USA. The remaining authors declare that the research was conducted in the absence of any commercial or financial relationships that could be construed as a potential conflict of interests.

## Abbreviations

RCC: renal cell carcinoma
AUC: area under the curve
PCA: principal component analysis
OPLS-DA: orthogonal partial least squares discriminant analysis
CV: coefficient of variation
PC: glycerophosphocholines
PE: glycerophosphoethanolamines
SM: sphingomyelin
DG: diacylglycerol
PA: glycerophosphates
PI: glycerophosphoinositols
PS: glycerophosphoserines
NAD: nicotinamide-adenine dinucleotide.
