# Supplementary material for: Investigation of Plasma Metabolic and Lipidomic Characteristics of a Chinese Cohort and a Pilot Study of Renal Cell Carcinoma Biomarker
Source: Front Oncol. 2020 Aug 18;10:1507. doi: 10.3389/fonc.2020.01507 (PMC7461914; doi:10.3389/fonc.2020.01507)
Supplement: Supplementary file 1 [file Data_Sheet_1.docx]

Supplementary Materials

**Investigation of Plasma Metabolic and Lipidomic Characteristics of a Chinese Cohort and a pilot study of Renal Cell Carcinoma biomarker**

Xiaoyan liu^1,#^, Mingxin Zhang^2,3,#^, Xiang Liu^1,#^, Haidan Sun^1^, Zhengguang Guo^1^，Xiaoyue Tang^1^, Zhan Wang^2^, Jing Li^1^, Lu He^4^, Wenli Zhang^4^, Yajie Wang^5^, Hanzhong Li^2^, Lihua Fan^1^, Shirley X. Tsang^6^, Yushi Zhang^2,*^ , Wei Sun^1,*^

*^1^Institute of Basic Medical Sciences, Chinese Academy of Medical Sciences, School of Basic Medicine, Peking Union Medical College, Beijing, 100005, China*

*^2^Department of Urology, Peking Union Medical College Hospital, Chinese Academy of Medical Science, Beijing, China*

*^3^Department of Urology, The Affiliated Hospital of Qingdao University,Qingdao, China*

*^4^Beijing Tiantan Hospital, Capital Medical University, Beijing, China*

*^5^Core Laboratory for Clinical Medical Research, Beijing Tiantan Hospital, Capital Medical University, Beijing, China*

*^6^Principal Investigator BioMatrix Rockville, Maryland, USA*

^#^ These authors contributed equally to this work.

^*^ Corresponding author(s)

E-mail: [sunwei1018@sina.com](mailto:sunwei1018@sina.com) (Wei Sun), [zhangyushi2014@126.com](mailto:zhangyushi2014@126.com) (Yushi Zhang)

**Detailed Methods**

**Sample Preparation**

Metabolomics

50 µL of serum was diluted to 200 µL using H_2_O to reduce the adsorption loss of metabolites by protein. 400 µL of acetonitrile was added into serum sample, and vortexed for 1 min, stand for 60 min at -20 ºC and centrifuged at 14,000 × g for 10 min to remove serum proteins. The supernatant was dried under vacuum and then reconstituted with 100 μl of 2% acetonitrile. Serum metabolites were further separated from small protein molecules using 10 kDa molecular weight cut-off ultracentrifugation filters (Millipore Amicon Ultra, MA) before transferred to the autosamplers. The quality control (QC) sample was prepared by pooling two hundred representative samples across different groups to be analyzed and therefore globally representative of the whole sample set. The QC samples were injected every twenty samples throughout the analytical run to provide a set of data from which method stability and repeatability can be assessed.

Lipidomics

200 µL of serum samples were mixed with 600 ul of isopropanol (IPA) precooled to −20 °C. Samples were vortex mixed for 1 min. After 10 min of incubation at room temperature, samples were stored at −20 °C for 2 h to improve protein precipitation and then centrifuged at 14, 000g for 20 min. The supernatant was dried under vacuum and stored at –80ºC refrigerator until analysis. The dried lipids were reconstituted with 100 μl of 50% IPA. QC sample was prepared using the same strategy as metabolomics.

**LC-MS Analysis**

For metabolomics, metabolites were separated with a 18 min gradient on a Waters HSS C18 column (3.0 × 100 mm, 1.7 μm) at a flow rate of 0.5 ml/min. Mobile phase A was 0.1% formic acid in H2O and mobile phase B was acetonitrile. The gradient was set as follows: 0–1 min, 2% solvent B; 1–3min, 2–55% solvent B; 3–8 min, 55-100% solvent B; 8-13 min, 100% solvent B; 13-13.1 min, 100-2% solvent B; 13.1-18 min, 2% solvent B. The column temperature was set as 50℃.

For lipidomics, mobile phase A was 0.1% formic acid in H2O and mobile phase B was acetonitrile. The gradient was set as follows: 0 min, 40% solvent B; 0-2 min, 40-43% solvent B; 2-8 min, 43-85% solvent B; 8-15 min, 85-99% solvent B; 15-18 min, 99% solvent B; 18-18.1 min, 99-40% solvent B; 18.1-23 min, 40% solvent B. The column temperature was set as 55℃. Full MS acquisition scanned from 100 to 1000 m/z at a resolution of 60 K. Automatic gain control (AGC) target was 1× 10^6^ and maximum injection time (IT) was 100 ms. Subsequently differential metabolites identification was performed by UPLC targeted-MS/MS analyses of QC sample. It acquired at a resolution of 15 K with AGC target of 5× 10^5^, maximum IT of 50 ms, and isolation window of 3 m/z. Collision energy was optimized as 20, 40, 60 for each target with higher-energy collisional dissociation (HCD) fragmentation.

**Data processing using Progenesis QI**

The detailed workflow for data processing facilitated by Progenesis QI is involved “create a

new experiment”, “import data”, “review alignment”, “experiment design setup”, “peak picking”, “reviewed convolution”, and “identify compounds” in sequence. In general, the whole process ran automatically using optimized parameter settings. (1) In the stage of create a new experiment, adduct ion was carefully selected as it would influence the number of characterized compounds and also the identification accuracy. Based on the ionization behaviors of reference standards, the adduct ion forms, comprising [M + H]+, [M + Na]+, [M + K], [M + NH4]+, [2M + H]+, [2M + Na]+,[2M + NH4]+, [M + H – H2O]+ and [M + H – 2H2O]+ , were selected. (2) The MS data acquired by LC-MS for all the plasma samples were imported into the Progenesis QI software, generating a 2D ion intensity map with the retention time and m/z information as the ordinate and abscissa, respectively. (3) Peak alignment was carried out in automatic manner taking a QC run as the reference, the score values for all the samples were greater than 90 %. (4) For peak picking, the thresholds of chromatographic peak absolute intensity, and retention time limits can be set to achieve the maximum real ion signals with noise excluded. In the present study, absolute intensity and retention time limit were set at 1000 and default. Peak intensity was normalized using log2 normalization of all the compound. (5) Further compound identification was performed by searching the HMDB database (2017 version). The identification results combined with the intensity data were exported as .csv files for subsequent compound confirmation and multivariate statistical analysis.

**Confirmation of compounds characterization**

Detailed compound identification information (.csv file) included compound ID, adducts, formula, score, fragmentation score, mass error (in ppm), isotope similarity, theoretical isotope distribution, web link, and m/z values. The data was further analyzed in detail, under which more abundant MS/MS fragments were acquired. Confirmation of the differential compounds was performed by the parameters, including Score, Fragmentation score, and Isotope similarity given by Progenesis QI. Score ranging from 0 to 60, is used to quantify the reliability of each identity. According to the score results of the reference standards, the threshold was set at 35.0. Fragmentation score represents the matching degree between the theoretical fragments and the measured ones. The fragmentation score of 0 indicates no match occurs or the compound generates no fragments. Isotope similarity is calculated by comparison of the measured isotope distribution of a precursor ion with the theoretical. The compound identification is more reliable the higher the values obtained.

**Pathway analysis**

Mummichog is a program written in python for analyzing data from high‐throughput, untargeted HRLC‐MS metabolomics, bypassing the tedious and challenging metabolite identification. It leverages the organization of metabolic networks to predict functional pathways directly from feature tables and generate a list of tentative metabolites annotations through functional activity analysis. We input tab‐delimited text files of peaks list with m/z, P value, and fold change of two group analysis into Mummichog to conduct the pathways and module analysis. KEGG human network model was selected, and the cut‐off P value was set to 0.05 to generate a list of significant features. The analytical mode of mass spec was set to positive according the data source. Other options remained the default. Results from annotation, pathway analysis, and network module analysis were given. A p value for each pathway was given to show the confidence of the results. We then used MetaboAnalyst (http://www.metaboanalyst.ca/) to visualize the results files of the metabolic pathways network.

**Results**

**Quality control**

The large cohort of samples was analyzed randomly. QC is of importance in large-scale metabolomics studies to ensure stable system performance and limit experimental bias. A pooled mixture of aliquots from representative serum samples in each group was prepared as a QC standard. The QC sample was injected 5 injections before and frequently throughout the analytical run to monitor instrument stability. Overall 27 injections for metabolomics and 27 injections for lipidomics were performed during the whole analysis. Variation of QC injections with analysis time was evaluated to assess the technical reproducibility. Variation of lipidomics QC injections is larger than metabolomics, although both analysis platforms showed acceptable stability and reproducibility (< ± 2SD)(Fig.S). The results provided some assurances that the platform had essential repeatability and stability throughout the analytical run.

a


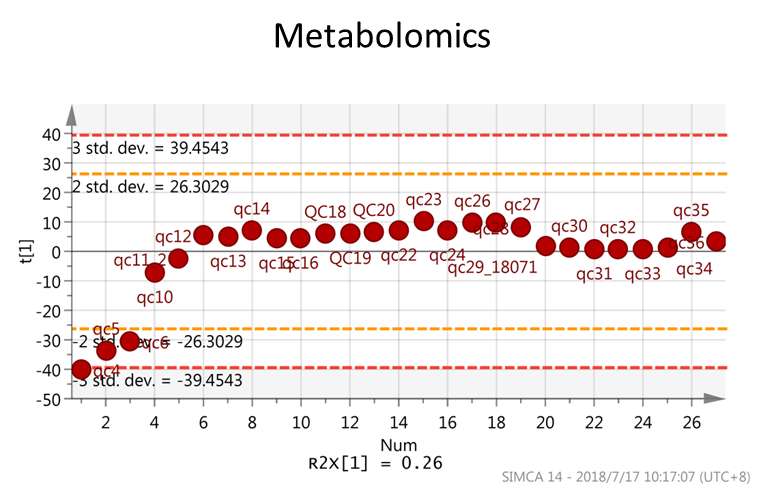


b


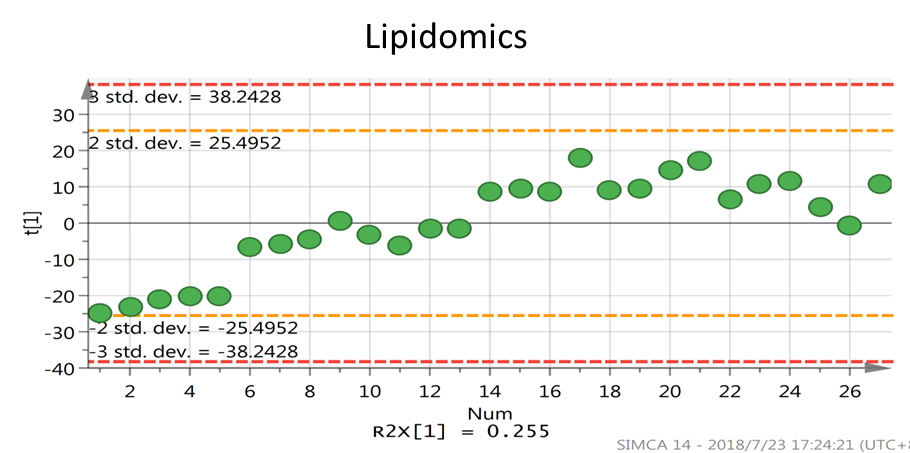


Fig. S Stability of QC samples of serum metabolomics and lipidomics. Variation of the first component was within ± 2SD, indicated good stability of the QC samples.
