# Supplementary material for: Investigation of Plasma Metabolic and Lipidomic Characteristics of a Chinese Cohort and a Pilot Study of Renal Cell Carcinoma Biomarker
Source: Front Oncol. 2020 Aug 18;10:1507. doi: 10.3389/fonc.2020.01507 (PMC7461914; doi:10.3389/fonc.2020.01507)

Supplementary Figures

Xiaoyan liu^1,#^, Mingxin Zhang^2,3,#^, Xiang Liu^1,#^, Haidan Sun^1^, Zhengguang Guo^1^，Xiaoyue Tang^1^, Zhan Wang^2^, Jing Li^1^, Lu He^4^, Wenli Zhang^4^, Yajie Wang^5^, Hanzhong Li^2^, Lihua Fan^1^, Shirley X. Tsang^6^, Yushi Zhang^2,*^ , Wei Sun^1,*^

*^1^Institute of Basic Medical Sciences, Chinese Academy of Medical Sciences, School of Basic Medicine, Peking Union Medical College, Beijing, 100005, China*

*^2^Department of Urology, Peking Union Medical College Hospital, Chinese Academy of Medical Science, Beijing, China*

*^3^Department of Urology, The Affiliated Hospital of Qingdao University,Qingdao, China*

*^4^Beijing Tiantan Hospital, Capital Medical University, Beijing, China*

*^5^Core Laboratory for Clinical Medical Research, Beijing Tiantan Hospital, Capital Medical University, Beijing, China*

*^6^Principal Investigator BioMatrix Rockville, Maryland, USA*

*Corresponding author: Prof. Wei Sun, E-mail: sunwei1018@sina.com; Tel.: 0086-010-69156995

Prof. Yushi, Zhang, E-mail: zhangyushi2014@126.com; Tel.: 0086-010-69152529

^#^ These authors contributed equally to this work.

**Fig S1** Individual variations of plasma metabolomics and lipidomics in normal subjects with different gender and age.

**
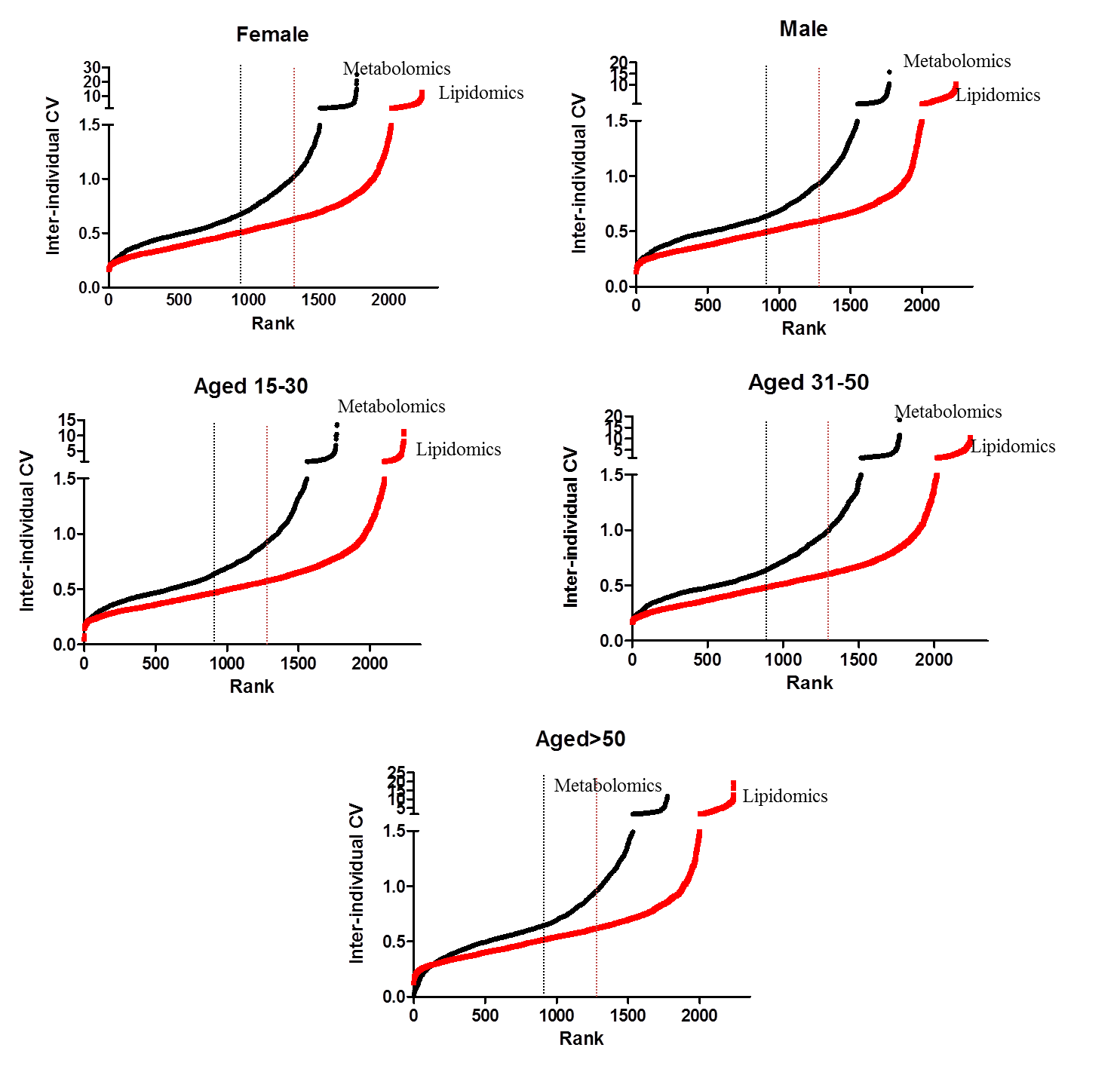
**

**Fig S2** Analysis of serum metabolic profiling variation of gender. a. Score plot of unsupervised PCA overview of serum metabolic profiling between male and female. b. Permutation validation of OPLS-DA model. c. Relative intensity of differential metabolites with gender and the enriched pathway these metabolites involved in.


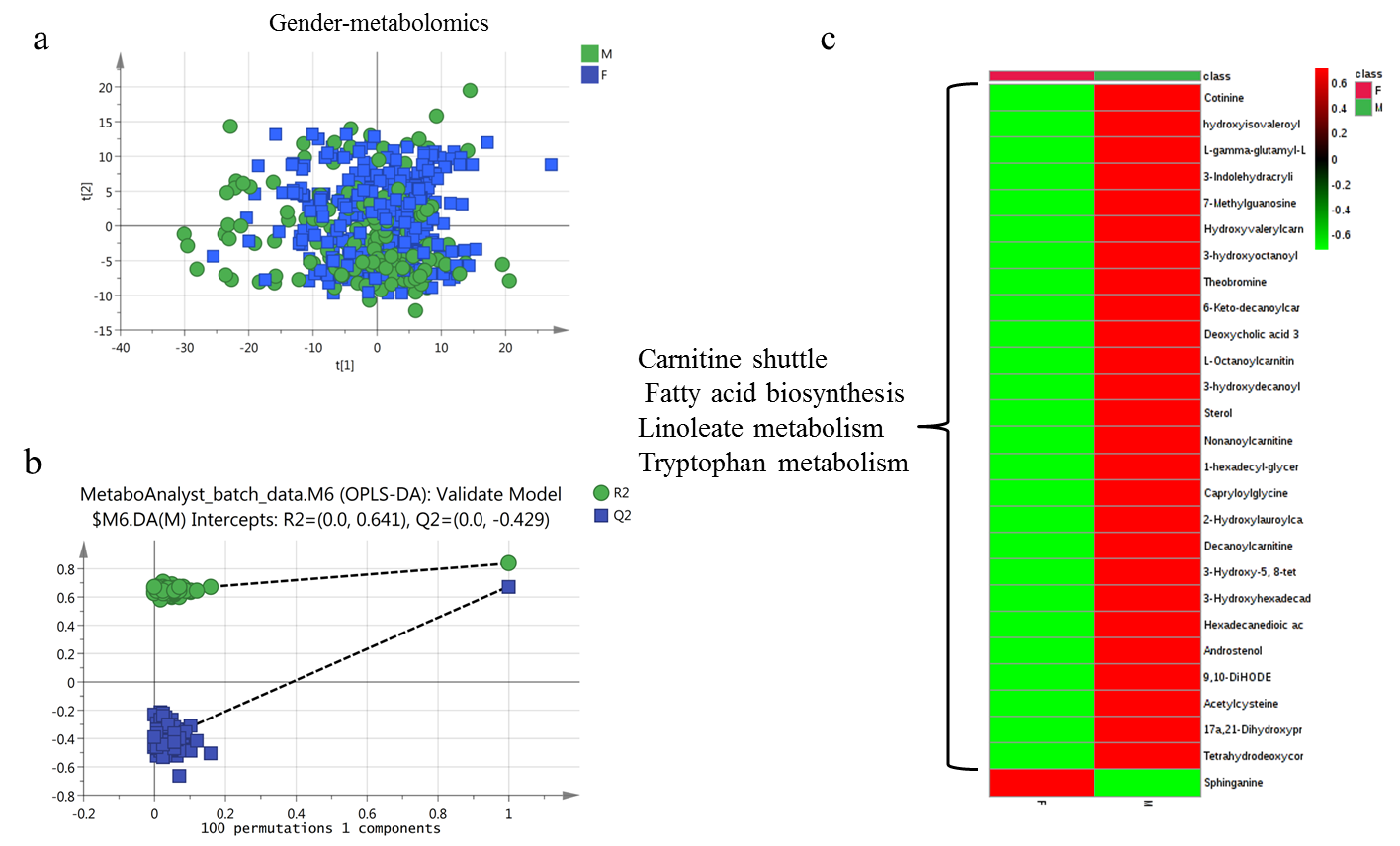


**Fig S3** Analysis of serum lipidomics variation of gender. a. Score plot of unsupervised PCA overview of serum lipidomics between male and female. b. Score plot of OPLS-DA model for serum lipidomics between male and female. c. Permutation validation of OPLS-DA model . d. Relative intensity of differential lipids with gender and the main pathway these lipids involved in.


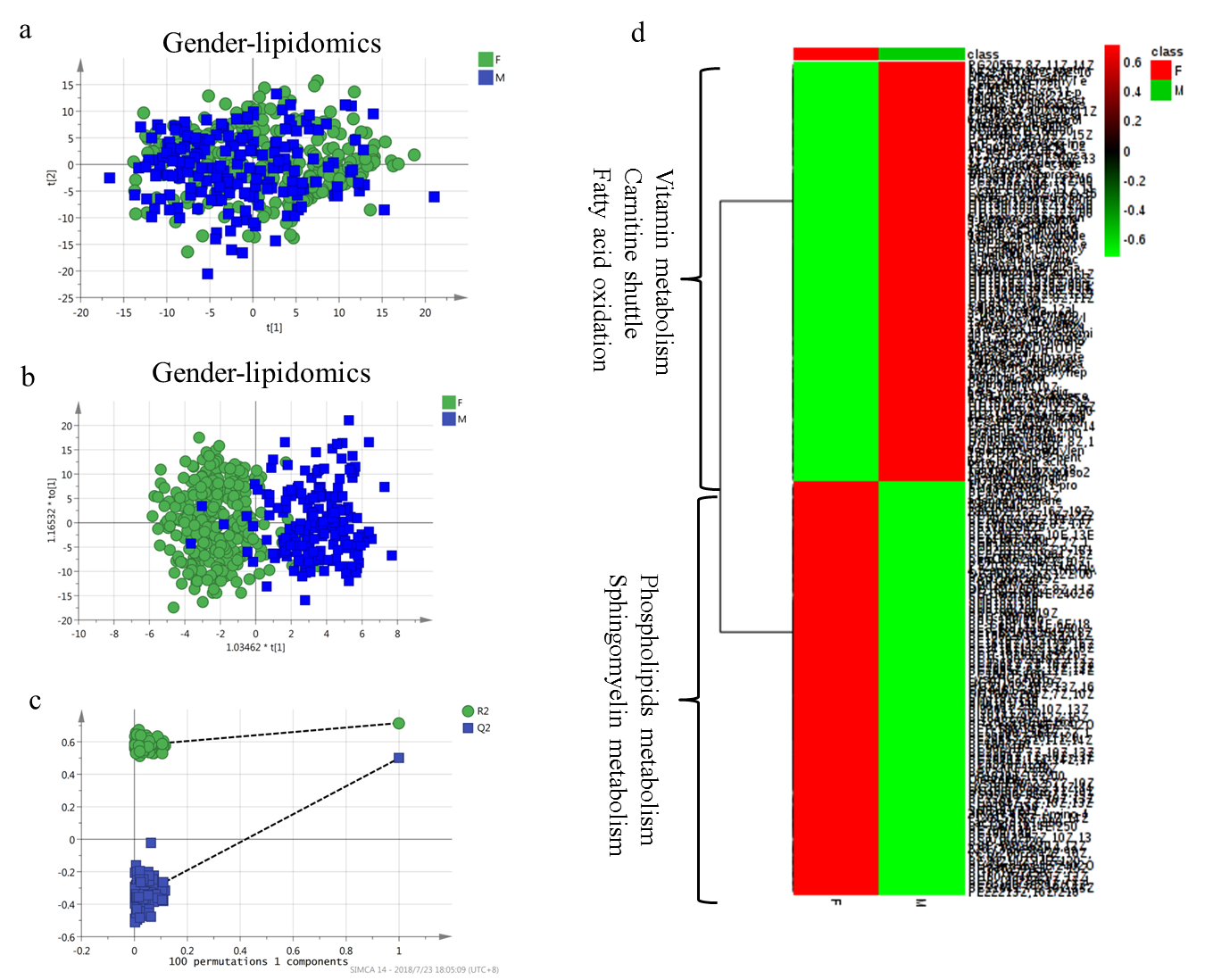


**Fig S4** PCA Score plot of three age stages based on serum metabolomics and lipidomics in female and male.


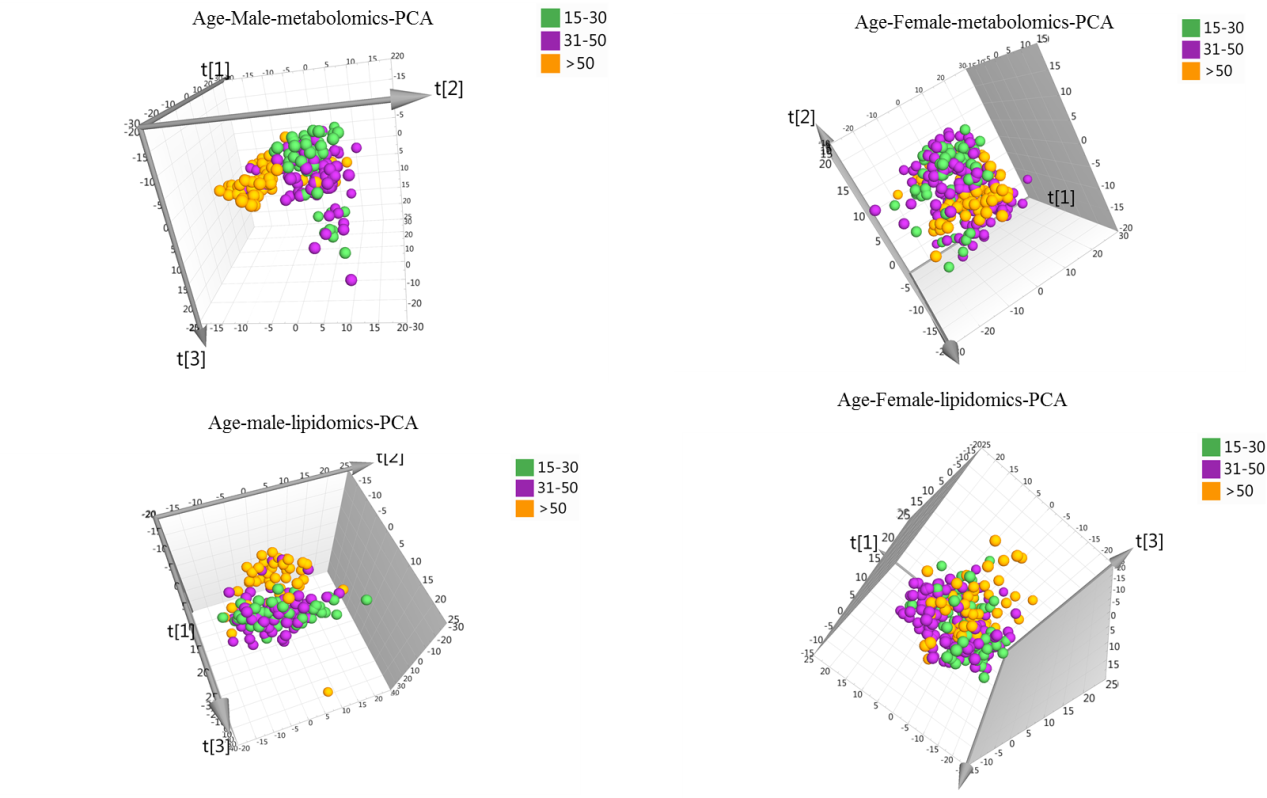


**Fig S5** **a,b.** Score plot of PLS-DA model for three age stages based on serum lipidomics in female (a) and male (b). **c.d.** Differential lipids pathways with respect to age in females (c) and males (d)


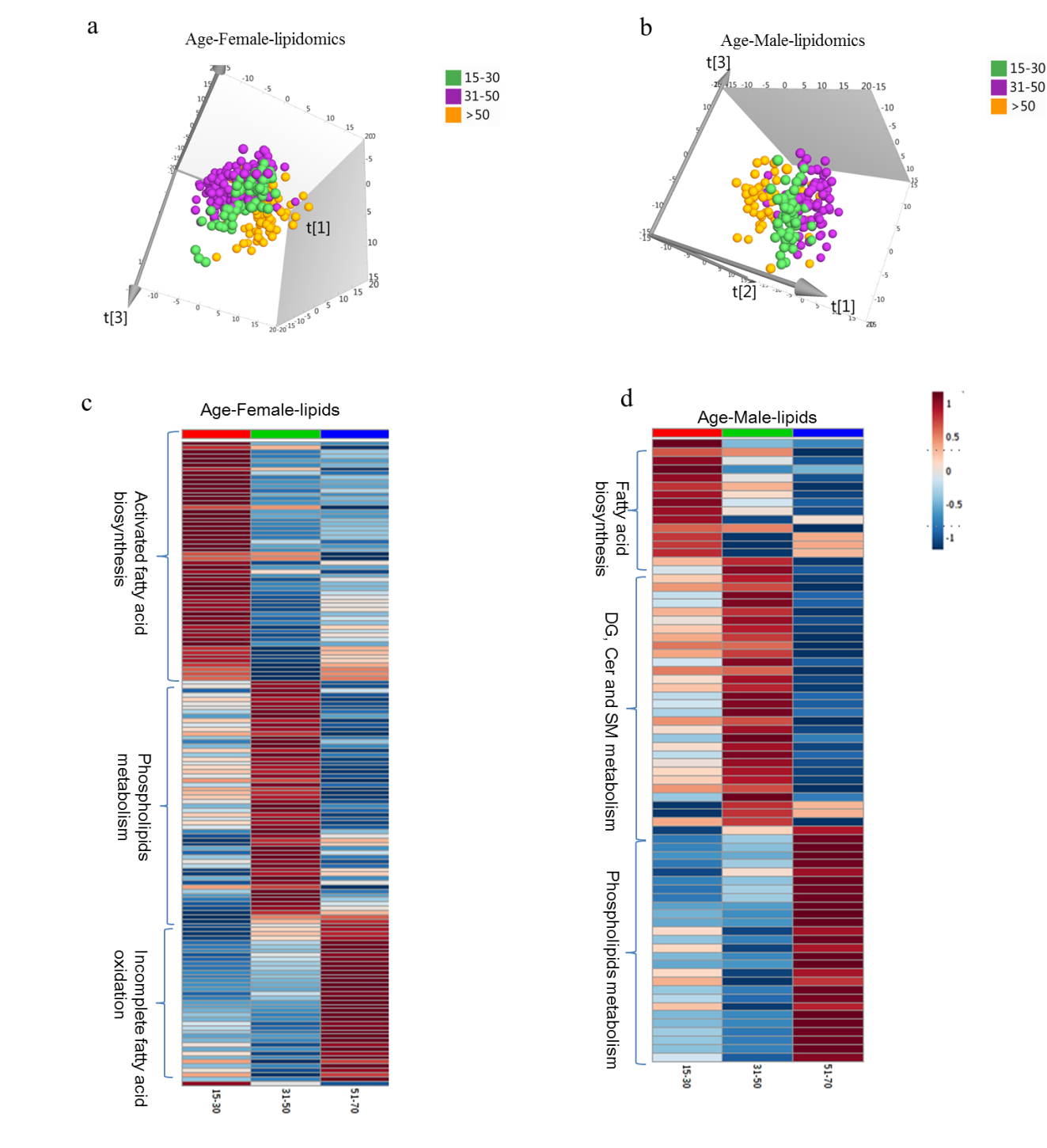


**Fig S6** Analysis of metabolomics and lipidomics variation between RCC and health control. a. PCA score plot of metabolomics between RCC and health control group. b. PCA score plot of lipidomics between RCC and health control group. c. OPLS-DA score plot of lipidomics between RCC and controls.


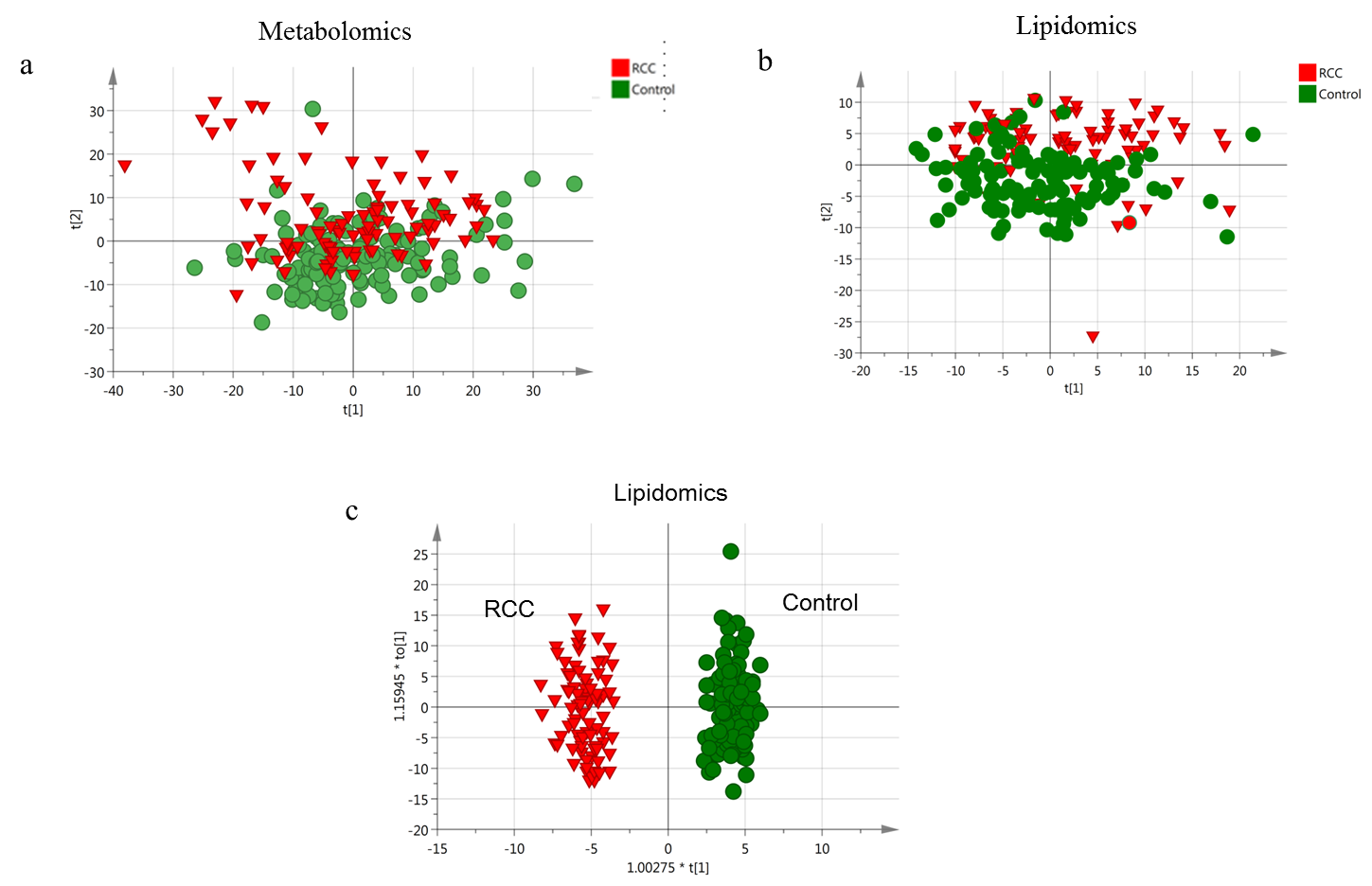

Supplement: Supplementary file 2 [file Data_Sheet_2.docx]
